# Advances and frontiers in pulmonary fibrosis and lung cancer research (2000–2024): a bibliometric analysis

**DOI:** 10.3389/fmed.2025.1596228

**Published:** 2025-06-23

**Authors:** Yu Jiang, Jianjian Yu, Lina Fu, Yuxia Liu, Shitao Li, Yu Ding, Hui Li, Chengsen Cai, Jun Wang

**Affiliations:** ^1^College of First Clinical Medicine, Shandong University of Traditional Chinese Medicine, Jinan, Shandong, China; ^2^Department of Respiratory and Critical Care Medicine, The Second Affiliated Hospital of Shandong University of Traditional Chinese Medicine, Jinan, Shandong, China

**Keywords:** pulmonary fibrosis, lung cancer, VOSviewer, CiteSpace, visual analysis

## Abstract

**Background:**

Lung cancer is the most common cancer and the leading cause of cancer-related death worldwide. In pulmonary fibrosis (PF), the incidence of lung cancer is elevated, and its prognosis is worse compared to the general population. With the development of related research, the relationship between lung cancer and pulmonary fibrosis has received close attention. However, comprehensive and objective reports on this topic remain scarce. Therefore, this study aims to identify research hotspots and visualize evolving trends and collaboration networks in the field of pulmonary fibrosis and lung cancer using bibliometric and knowledge mapping tools.

**Methods:**

Articles in the field of pulmonary fibrosis and lung cancer were retrieved using the Web of Science core collection subject search, and bibliometric analysis was performed in CiteSpace, VOSviewer, ChiPlot (https://www.chiplot.online/) and Bibliometrix (R-Tool of R-Studio).

**Results:**

This bibliometric analysis included 1,830 publications from 2000 to 2024, showing a steady increase over time. Collaborative network analysis identifies Japan, the United States, and China as the most influential countries, contributing the highest publications and citations. Respiratory Research is the leading journal. Bade BC is a key author, with Lung Cancer 2020: Epidemiology, Etiology, and Prevention as the most cited work. Literature and keyword analyses indicate a primary focus on diagnosis and survival, with recent shifts toward gene regulation and pulmonary inflammation. Emerging research highlights epithelial-mesenchymal transition (EMT) and chronic inflammation in lung cancer development among IPF patients. Notably, studies on immune checkpoint inhibitors (e.g., PD-1/PD-L1) have surged, reflecting a growing interest in immunotherapy.

**Conclusion:**

This study is the first to employ bibliometric methods to visualize research trends and frontiers in pulmonary fibrosis and lung cancer. Our analysis reveals a shift from early studies on diagnosis and prognosis toward a growing focus on molecular mechanisms and immunotherapy. These findings offer valuable insights into emerging research directions and may serve as a reference for researchers seeking to identify key topics and potential collaborators.

## 1 Introduction

Pulmonary fibrosis (PF) is a chronic and progressive interstitial lung disease that encompasses a spectrum of etiologies, including autoimmune, environmental, and idiopathic origins. Among these, idiopathic pulmonary fibrosis (IPF) is the most extensively studied subtype. It is a chronic, progressive fibrosing lung disease of unknown etiology (1). The median survival after diagnosis is approximately 3.5 years (2–4). Histologically, IPF is characterized by a usual interstitial pneumonia (UIP) pattern, which includes reticular abnormalities, traction bronchiectasis, and honeycombing (2, 5–8). Lung cancer (LC) remains the leading cause of cancer death worldwide and is responsible for over 1.6 million deaths per year (9). This accounts for approximately 20% of all cancer deaths, and is more than the combined total for breast, colon and prostate cancer. (10) The association between PF and LC cannot be ignored, as PF is considered a precancerous condition (11). Even after accounting for the impact of smoking, pulmonary fibrosis—especially idiopathic pulmonary fibrosis (IPF)—remains significantly associated with a 3.5–7.3-fold increased risk of lung cancer, particularly in Asian populations. Advanced age, male sex, and smoking are independent risk factors for malignant transformation in pulmonary fibrosis (12), with an even higher risk observed in individuals with concomitant pulmonary fibrosis and emphysema (13). Although the underlying pathogenic mechanisms remain unclear, studies suggest that they share certain common pathways (14), including tobacco exposure, genetic and epigenetic alterations, aging, and pathological epithelial-mesenchymal crosstalk. Bibliometric analysis is a method that uses mathematical and statistical methods to review and analyze studies in a specific field of research over a specific period, both qualitatively and quantitatively (15). This method focuses on countries, institutions, journals, authors, and keywords related to research in a specific field, providing readers with an objective view of trends and frontiers in the field (16, 17). Bibliometric analysis has been used in many research areas, including Neurology (18), kidney disease (19), periodontology (20), Pharmacological Research (21) and other areas. Despite the rapid development of PF and LC related research over the past 25 years, there is still a lack of bibliometric analyses related to the field of PF and LC. Therefore, this study aims to visually and quantitatively analyze the research landscape of PF and LC from 2000 to 2024, with a focus on identifying research hotspots, collaboration patterns, and emerging trends. We employed two bibliometric tools, VOSviewer and CiteSpace, to provide a comprehensive reference for researchers seeking to understand the field and identify potential collaborators.

## 2 Materials and methods

### 2.1 Data sources and search strategies

We used the SCI-expanded of WoSCC bibliographic database developed by Thomson Scientific to perform bibliometric analysis (22). Considering rapid database renewal, literature retrieval was conducted on a single day (January 12, 2025) to avoid deviations. The publication period in this study was set between 2000 and 2024. The search terms were presented as follows:

1: TS = (“Pulmonary Fibrosis” OR “Pulmonary Fibroses” OR “Fibrosing Alveolitides” OR “Fibrosing Alveolitis” OR “Idiopathic Diffuse Interstitial Pulmonary Fibrosis”)
2: TS = (“Lung Neoplasm*” OR “Lung Cancer*” OR “Pulmonary Neoplasm*” OR “Cancer of Lung” OR “Pulmonary Cancer*” OR “Cancer of the Lung”)
3: #1 AND #2

**FIGURE 1:**
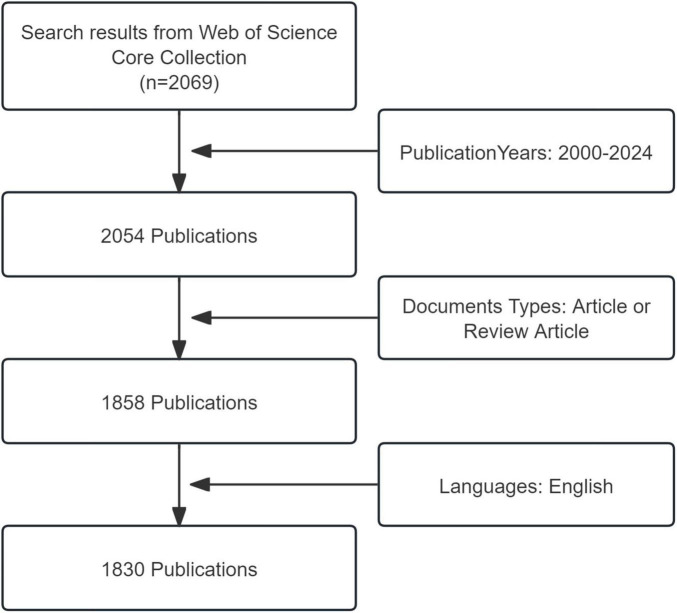
Flowchart of the literature screening process.

Only English articles and reviews were included in the analysis, while other forms of publications, such as reprints, book chapters, conference abstracts, news items, letters, editorial material, corrections, data files, early access, bibliographies, and biographical entries were excluded. Duplicate publications were identified and removed using Citespace. A total of 1,830 publications were collected and analyzed (Figure 1).

### 2.2 Data analysis and visualization

CiteSpace, developed by Chaomei Chen, is currently the most widely used software for bibliometric analysis (23). We used CiteSpace.6.4.R1 Advanced visualization to analyze country distribution and collaboration, the dual-map overlay of journals, institutional distribution, subject area distribution, keyword timeline graphs, reference collaboration and literature bursts. VOSviewer was developed by Nees Jan van Eck et al. And is mainly used for bibliometric network graph analysis (24). We used VOSviewer 1.6.20 to visually analyze country distribution, institution distribution, author distribution and collaboration and keyword collaboration. The clustering, which relies on the similarity matrix and VOS mapping technique, was completed automatically and the corresponding labels were then added by the authors according to the content. In addition, we used Bibliometrix (R-Tool of R-Studio) to visually analyze the country distribution, references and keywords (25), and ChiPlot^1^ to show the publications count of the literature over the years. Finally, to analyze the trend of PF and LC, curve fitting was performed using R (version 4.4.1; R Core Team (25)).

## 3 Results

### 3.1 Annual publications and citation trends

**FIGURE 2:**
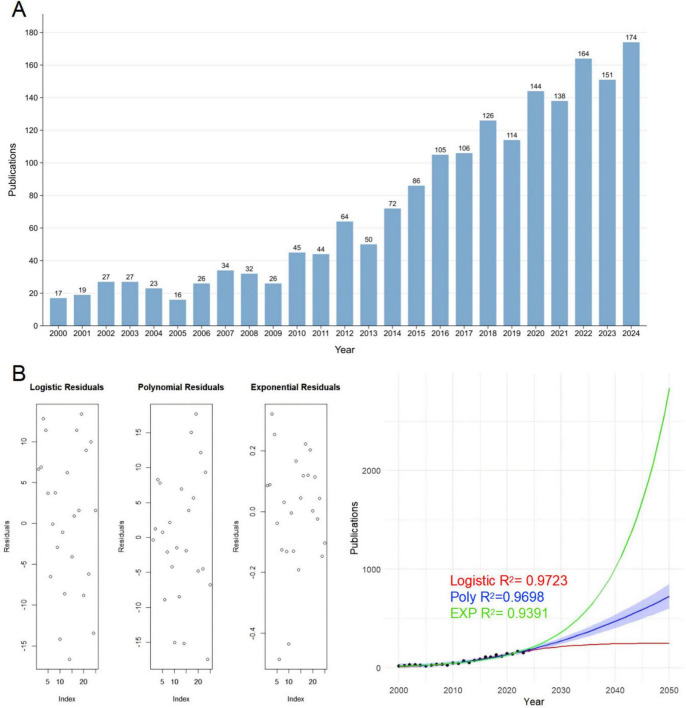
Description of publication volume and forecast of publication volume. **(A)** Annual publication count on pulmonary fibrosis and lung cancer (2000–2024), showing an overall upward trend, peaking at 174 in 2024. The count fluctuates between 16 and 174, with a 10-year average (2015–2024) exceeding 130. **(B)** Predicted publication trends for 2025–2050. The red, blue, and green curves represent logistic, polynomial (degree = 2), and exponential regression models, respectively. The blue shaded area indicates the confidence interval of the polynomial model. R2 represents the goodness of fit, where a value closer to 1 indicates better model performance. The left panel shows the residual distribution.

Figure 2A shows the annual publication trends in PF and LC from 2000 to 2024. From 2000 to 2014, publications fluctuated between 20 and 40 per year, with a low of 16 in 2004. Since 2015, publications have increased significantly, rising from 72 in 2015 to 174 in 2024, indicating growing interest in this field.

This increase may be related to several factors. In 2015, immune checkpoint inhibitors like nivolumab and pembrolizumab were approved as second-line treatments for advanced non-small cell lung cancer, marking a shift in lung cancer therapy. At the same time, antifibrotic drugs such as nintedanib and pirfenidone made clinical progress, offering new treatment options. These developments likely encouraged research into combining immunotherapy and antifibrotic treatment in patients with both conditions. Additionally, molecular studies suggest the fibrotic microenvironment may promote tumor metastasis via specific signaling pathways, further driving research interest. In summary, the rise in publications after 2015 likely reflects advances in immunotherapy, antifibrotic therapy, and molecular research, making this a hot topic in academia.

Figure 2B shows logistic regression, quadratic polynomial regression, and exponential regression models by R-Studio (the right one). To evaluate the model fit, residual distributions were examined (the left one). The residuals of the logistic and polynomial models exhibit greater fluctuations, whereas the residuals of the exponential model are relatively smaller but more concentrated. This suggests that while the exponential model may fit the data well in the short term, it might carry the risk of overfitting. To further compare the prediction trends of the three models, the right one presents the fitted publication trends over time. The logistic model (red curve) aligns well with actual data in the short term but plateaus in long-term predictions, indicating a saturation effect. The exponential model (green curve) demonstrates an exponential growth pattern; however, its long-term predictions appear overly aggressive, leading to potential overestimation. In contrast, the polynomial model (blue curve) provides a relatively smoother trend, and the blue shaded area represents its confidence interval, reflecting the range of uncertainty in predictions. Therefore, in forecasting publication trends, the polynomial regression model may serve as a balanced approach, capturing past growth trends without over-exaggerating future expansion. Its confidence interval suggests that by 2,050, the annual publication count will range between 800 and 1,200 papers, representing a steady increase but at a slower rate compared to the exponential model. This implies that research on PF and LC is likely to continue growing in the coming decades but may experience a gradual deceleration.

### 3.2 Distributions of countries/regions

**FIGURE 3:**
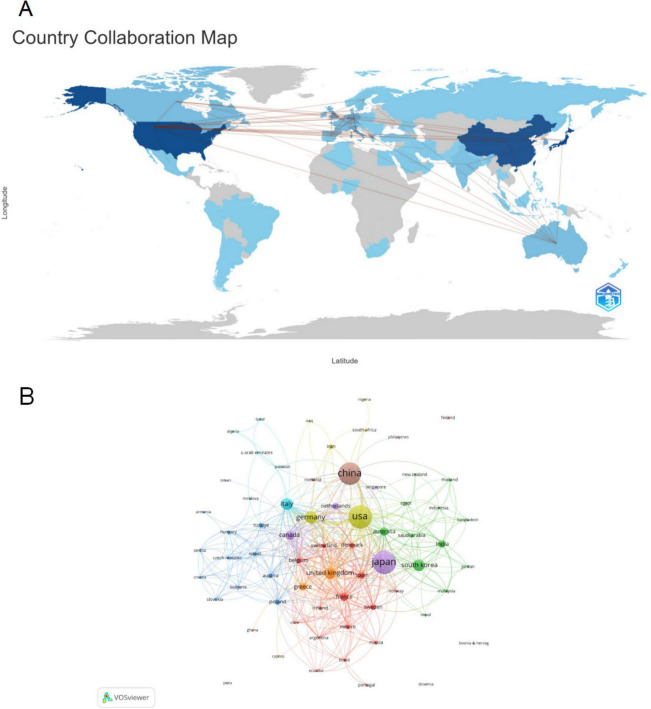
Country/region analysis of pulmonary fibrosis and lung cancer research. **(A)** Countries/regions involved in the research, with links indicating collaboration. The thickness of the links indicates the strength of collaboration between countries/regions. **(B)** VOSviewer visualization of the collaboration network, showing countries/regions with > 1 publication. Different node colors represent distinct clusters, and node size is proportional to the number of publications.

Currently, 66 countries/regions are involved in research on PF and LC, with a predominant focus in the Northern Hemisphere. Notably, inter-country/regional collaborations are primarily concentrated among North America, Europe, East Asia, and Oceania, forming strong connections (Figure 3A). Table 1 lists the top 10 countries/regions based on publication volume, with their corresponding citation frequency and centrality values. The betweenness centrality of a country/region reflects its importance in the network. Japan has the highest number of publications (441), followed by the United States (435) and China (402). The United States has the highest citation frequency (29,249), followed by Japan (14,004). The citation frequency of all other countries/regions is below 10,000. The relatively high publication output from Japan likely reflects the country’s strong research activity and accumulated expertise in the field of PF and LC. The United States exhibits the highest citation frequency, suggesting that its published studies tend to have greater academic impact and contribute significantly to the advancement of this research area.

**TABLE 1:** Top 10 countries/regions ranked by number of publications, with corresponding citation frequencies and centrality.

Countries/regions	Publications	Citations	Centrality
Japan	441	14,004	0
USA	435	29,249	0.19
China	402	7,760	0.07
Germany	115	6,318	0.1
Italy	109	3,833	0.17
South Korea	104	3,585	0.02
United Kingdom	99	6,404	0.33
Canada	57	3,328	0.21
France	54	3,565	0.03
Australia	51	3,390	0.14

Figure 3B and Supplementary Figure 1 illustrates the international collaboration among the countries. The global collaboration network analysis reveals that countries and regions are roughly divided into 12 clusters in VOSviewer, distinguished by different colors based on the strength of their collaboration (Figure 3B). In CiteSpace, each node represents a country/region, with the radius of the node increasing proportionally to its contribution to PF and LC research (Supplementary Figure 1). The links between nodes indicate collaborative relationships between individual countries and regions, with the thickness of the links positively correlated with the depth of collaboration. The betweenness centrality of a node reflects its connectivity with other nodes and is proportional to the size of the surrounding purple ring. A larger purple ring indicates a higher betweenness centrality value. The United Kingdom and Canada are major research hubs for PF and LC, maintaining close collaborations with Germany, Italy, Australia, and other countries. International collaboration networks highlight the essential role of cross-country partnerships in promoting knowledge exchange and driving innovation within the field.

### 3.3 Analysis of institution

The analysis of research institutions aims to understand the global distribution of studies related to PF and LC and to identify potential collaboration opportunities. Table 2 lists the top 10 institutions based on publication volume, with their corresponding citation frequency and centrality values. Harvard University has the highest number of publications (39), followed by the University of California System (38). Among the top 10 institutions in terms of publication volume, four are from Japan, two from the United States, and two from China. Notably, Royal Brompton Hospital (0.14), Assistance Publique Hopitaux Paris (APHP) (0.11), Seoul National University (SNU) (0.11), and several other institutions exhibit high centrality (Supplementary Table 1), indicating their significant role in research on PF and LC.

**TABLE 2:** Top 10 institutions by number of publications, with corresponding centrality and citation.

Rank	Institution	Publications	Centrality	Citation
1	Harvard University	39	0.09	2,633
2	University of California System	38	0.09	3,370
3	National Cancer Center—Japan	32	0.04	1,769
4	Kanagawa Cardiovascular & Respiratory Center	24	0.05	589
5	Juntendo University	24	0	186
6	Huazhong University of Science & Technology	23	0.02	786
7	Helmholtz Association	23	0.03	1,735
8	Assistance Publique Hopitaux Paris (APHP)	22	0.11	838
9	Sichuan University	21	0	336
10	Hiroshima University	21	0	290

**FIGURE 4:**
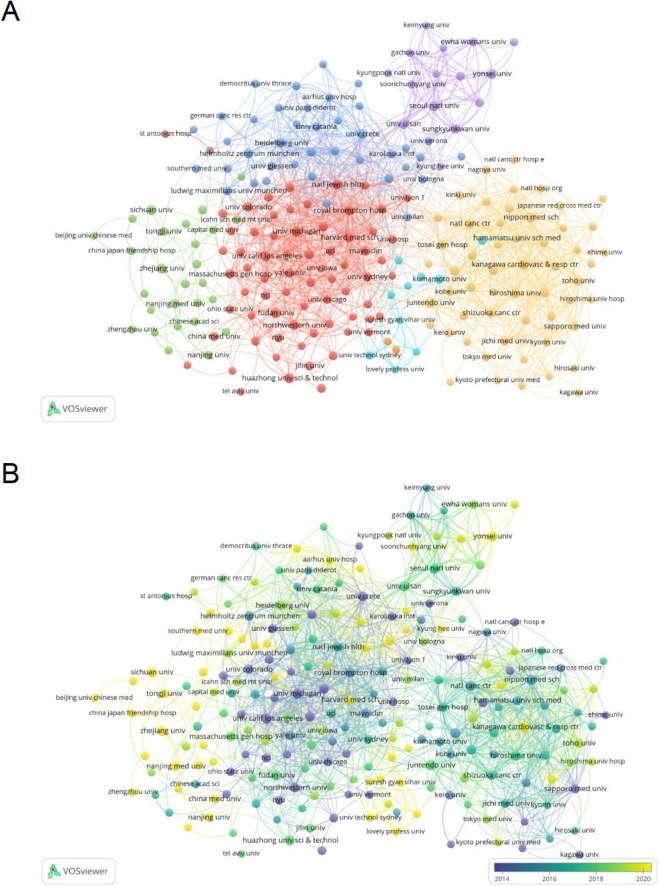
Institutional analysis of pulmonary fibrosis and lung cancer research. **(A)** VOSviewer visualization of the institutional collaboration network, showing institutions with > 5 publications. Different node colors represent distinct collaboration clusters, and node size indicates publication count (occurrence frequency), reflecting the relative research output of each institution. Links between nodes illustrate collaborative relationships, with thicker links indicating stronger cooperation. **(B)** Visualization of institutional publication activity over time, where node color represents the average publication year (APY). Warmer colors (e.g., yellow) indicate more recent research activity, while cooler colors (e.g., blue) denote earlier contributions, highlighting trends in emerging versus established institutional involvement.

In VOSviewer, institutional collaborations are grouped into eight closely related clusters (Figure 4A). Figure 4B illustrates the distribution of institutional publication activity over time. The color of each node represents the average publication year (APY), calculated as the sum of the publication years of all papers associated with an institution divided by its total number of publications. A higher APY (yellow) indicates emerging contributors in PF and LC research, whereas a lower APY (blue) suggests institutions with fewer recent publications. The results highlight a significant increase in research output over the past 5 years from institutions such as Harvard Medical School, Zhejiang University, and Zhengzhou University. In contrast, institutions like the University of Michigan and Yale University have shown relatively lower engagement in this field during the same period. In CiteSpace, Harvard University appears as the most productive institution within the collaboration network, yet its centrality is lower than that of Royal Brompton Hospital, indicating that the latter plays a more central role in institutional collaboration networks (Supplementary Figure 2).

### 3.4 Analysis of authors

Co-citation author analysis refers to the phenomenon where two authors are simultaneously cited by a third author. A higher co-citation frequency indicates a closer academic interest and research density (26). Analyzing the most frequently cited authors in studies related to PF and LC, in terms of publication count and co-citation frequency, provides an intuitive reflection of the authors’ research influence and key research topics in this field.

**FIGURE 5:**
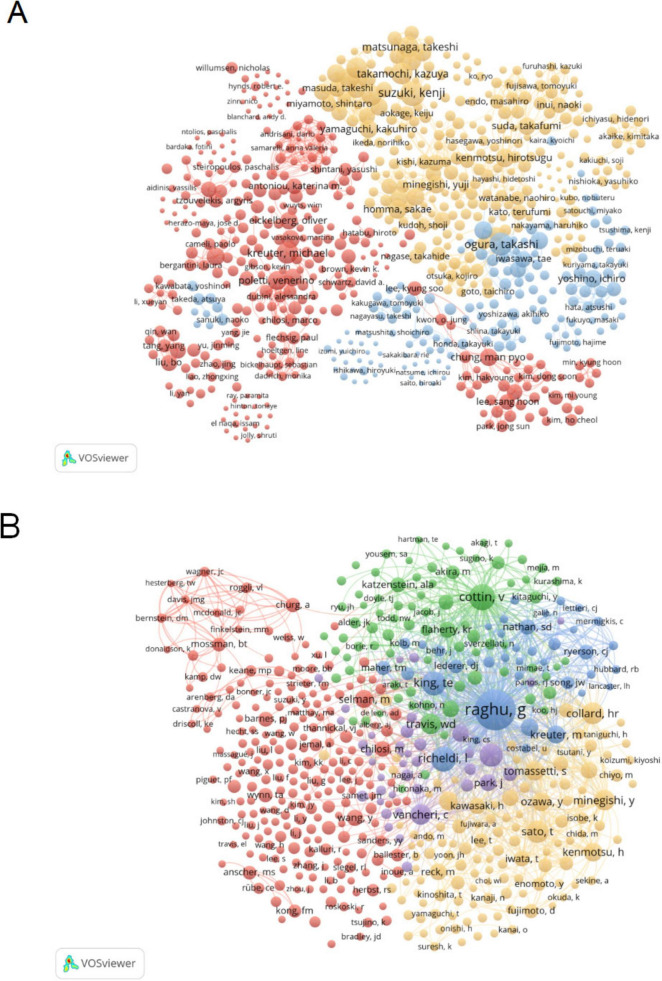
Analysis of author in pulmonary fibrosis and lung cancer research. **(A)** Visual representation of the author collaboration network in VOSviewer, where node colors indicate different clusters, and node size reflects occurrence frequency. **(B)** Visualization of the co-citation network among authors in VOSviewer, with node size representing occurrence frequency. The co-citation network reveals major author groups with high academic influence and thematic consistency, indicating dominant research directions and frequently cited key contributors in the field.

Table 3 and Supplementary Table 2 present the top 10 authors ranked by publication count and co-citation frequency, respectively, based on VOSviewer analysis, along with their total link strength. It includes the total number of publications, co-citation frequency, and the total link strength associated with each author. The most prolific author is Suzuki Kenji (20 publications), followed by Ogura Takashi (17 publications) and Takamochi Kazuya (16 publications). The most frequently co-cited author is Raghu G (988 co-citations), followed by Cottin V (343 co-citations) and Richeldi L (319 co-citations). The collaboration network of authors in PF and LC research is visualized in VOSviewer (Figure 5A), providing valuable insights for identifying potential research collaborators and field experts. Ogura Takashi and Kreuter Michael are central figures in the collaboration network. Among them, Ogura Takashi has established close collaborations with Gemma Akihiko, Hamada Hironobu, and Yamaguchi Kakuhiro, while Kreuter Michael works closely with Poletti Venerino. These key authors and their strong collaborative relationships highlight the high level of interaction in this research domain.

**TABLE 3:** Top 10 authors ranked by number of publications, with corresponding citations and total link strength.

Rank	Author	Publications	Citations	Total link strength
1	Suzuki Kenji	20	394	134
2	Ogura Takashi	17	415	115
3	Takamochi Kazuya	16	129	89
4	Kreuter Michael	15	850	48
5	Fukui Mariko	14	52	79
6	Hamada Hironobu	14	166	129
7	Matsunaga Takeshi	14	52	79
8	Okada Morihito	14	213	101
9	Hattori Noboru	13	165	128
10	Homma Sakae	12	258	47

The co-citation author network (Figure 5B) reveals a high degree of research homogeneity among scholars in the field of pulmonary fibrosis and lung cancer. The network is primarily divided into five major clusters, representing closely connected research communities: Raghu G, King TE, et al. (blue); Cottin V, et al. (green); Vancheri C, et al. (purple); Sato T, Minegishi Y, Collard HR, et al. (yellow); and Chilosi M, Selman M (red). These clusters indicate shared research interests and frequent co-citation patterns, suggesting thematic convergence and collaborative potential within each group.

### 3.5 Analysis of journals

**FIGURE 6:**
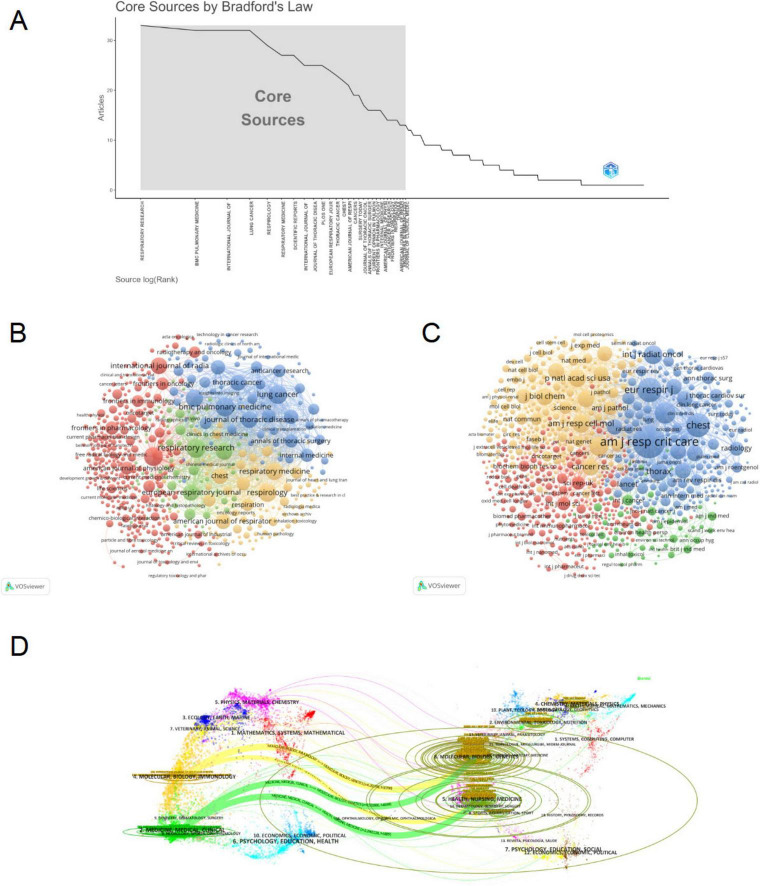
Journal analysis in pulmonary fibrosis and lung cancer research. **(A)** Bradford’s Law applies to academic journals in this field, with the gray-shaded area highlighting core journals ranked by publication volume. **(B)** Visualization of the journal co-occurrence network in VOSviewer, where node colors represent different clusters, and node size indicates occurrence frequency. **(C)** Visualization of the journal co-citation network in VOSviewer, with node size reflecting occurrence frequency. **(D)** Dual-map overlay of journals, where citing journals appear on the left, cited journals on the right, and colored paths represent citation relationships. The paths demonstrate citation trajectories between disciplines, indicating how research in pulmonary fibrosis and lung cancer integrates knowledge from multiple fields.

We utilized a bibliometric online analysis platform to identify high-impact journals with substantial publication output in the field of PF and LC. The impact factor (IF) and Journal Citation Reports (JCR) quartiles were used to assess journal influence. Journals ranking in the top 25% (including 25%) in terms of IF are classified as JCR Quartile 1 (Q1), while those ranking between 25 and 50% (including 50%) are classified as JCR Quartile 2 (Q2). Table 4; Supplementary Table 3 and Figure 6A show the top 10 journals based on publication count and co-citation frequency, with their corresponding impact factor (JCR 2023) and JCR quartile. The journal with the highest number of publications was Respiratory Research (IF 4.7, Q1, 33 articles), followed by BMC Pulmonary Medicine (IF 2.6, Q2, 32 articles), International Journal of Molecular Sciences (IF 4.9, Q1, 32 articles), and Lung Cancer (IF 4.5, Q1, 32 articles). Among the top 10 journals by publication volume, 7 journals were classified as Q1 JCR, and 5 had an IF exceeding 4. The most frequently co-cited journals were American Journal of Respiratory and Critical Care Medicine (IF 19.3, Q1, 5,260 co-citations), European Respiratory Journal (IF 17, Q1, 2,697 co-citations), and Chest (IF 9.5, Q1, 2,628 co-citations). Among the top 10 co-cited journals, 9 were classified as Q1 JCR, and 4 had an IF exceeding 10. Notably, International Journal Of Radiation Oncology Biology Physics and PLOS ONE, which ranked among the top 10 journals by publication volume, were also among the top 10 co-cited journals. The VOSviewer visualization illustrates the journals publishing PF and LC-related literature and their interconnections (Figure 6B). Clustering was based on journal similarity, forming four distinct groups. Red Cluster: This cluster primarily includes journals related to physiology, pharmacology, and cancer biology, such as Frontiers in Pharmacology, American Journal of Physiology, and Cytokine & Growth Factor Reviews. These journals focus on fundamental medical research, drug development, and physiological mechanisms. Blue Cluster: This group consists of journals specializing in thoracic diseases and oncology, including Journal of Thoracic Disease, Lung Cancer, and Thoracic Cancer, covering topics such as lung cancer, thoracic surgery, and radiotherapy. Yellow Cluster: This cluster comprises journals focusing on respiratory medicine, such as Respiratory Medicine, Respirology, and Chest, primarily addressing chronic respiratory diseases, pulmonary physiology, and clinical research. Green Cluster: This group includes journals emphasizing molecular biology and cancer mechanisms, with representative journals such as Molecular Cancer Research and Clinical Chimica Acta, which focus on molecular mechanisms of carcinogenesis and biomarker research. According to co-citation frequency, the journals were grouped into four clusters that share similar research orientations (Figure 6C). Blue Cluster: Primarily centered on pulmonary diseases and respiratory medicine, represented by journals such as American Journal of Respiratory and Critical Care Medicine. Red Cluster: Focused on oncology, including journals such as Cancer Research and International Journal of Molecular Sciences. Yellow Cluster: Encompassing fundamental biomedical research, with representative journals such as Journal of Biological Chemistry and Nature Medicine. Green Cluster: Concentrated on environmental and occupational health, represented by journals such as Environmental Health Perspectives. We used knowledge flow analysis to explore the evolution of knowledge citations and co-citation between citing and cited journals (27). The dual-map overlay of journals shows the topic distribution, changes in citation trajectories, and shifts in research centers across academic journals (Figure 6D) (27, 28). The labels on the left of the dual map represent citing journals, and the labels on the right represent cited journals. A colored curve of citation connections originating from the citing map and pointing to the cited map shows the context of the citation (27). The citing journals primarily belong to the fields of molecular biology, genetics, medicine, clinical research, health sciences, nursing, and psychology, representing the research frontiers in the domain. The cited journals predominantly originate from molecular biology, biochemistry, health sciences, dermatology, dentistry, surgery, mathematics, systems science, and computing, forming the knowledge base that underpins current research developments.

**TABLE 4:** Top 10 journals ranked by number of publications, with corresponding IF (JCR 2023) and JCR quartile.

Rank	Journal	Publications	IF (JCR2023)	JCR quartile	Total link strength
1	Respiratory research	33	4.7	Q1	277
2	BMC pulmonary medicine	32	2.6	Q2	310
3	International journal of molecular sciences	32	4.9	Q1	328
4	Lung cancer	32	4.5	Q1	479
5	Respirology	29	6.6	Q1	502
6	Respiratory medicine	27	3.5	Q2	389
7	Scientific reports	27	3.8	Q1	210
8	International journal of radiation oncology biology physics	25	6.4	Q1	191
9	Journal of thoracic disease	25	2.1	Q3	345
10	PLoS One	25	2.9	Q1	362

### 3.6 Analysis of keywords

Keywords provide a valuable lens to explore the evolving research frontiers in PF and LC. Table 5 lists the top 20 keywords by frequency, with e evolving research frontiers in (711) and “lung cancer” (710) as the most prominent, highlighting the central focus of this field. Other frequent keywords such as “survival” (208) and “acute exacerbation” (203) underscore the clinical challenges faced in managing these diseases.

**TABLE 5:** Top 20 keywords ranked by frequency of occurrence, with corresponding total link strength.

Rank	Keyword	Occurrences	Total link strength
1	Idiopathic pulmonary fibrosis	711	5,461
2	Lung cancer	710	5,278
3	Pulmonary fibrosis	430	3,089
4	Disease	240	2,047
5	Interstitial lung disease	226	1,947
6	Cancer	211	1,550
7	Survival	208	1,751
8	Acute exacerbation	203	1,847
9	Expression	194	1,462
10	Risk	171	1,356
11	Diagnosis	131	1,024
12	Cell lung-cancer	124	820
13	Non-small cell lung cancer	124	1,108
14	Chemotherapy	121	1,125
15	Mortality	120	992
16	Impact	118	1,031
17	Fibrosis	116	897
18	Inflammation	112	872
19	Risk-factors	98	873
20	Therapy	97	784

**FIGURE 7:**
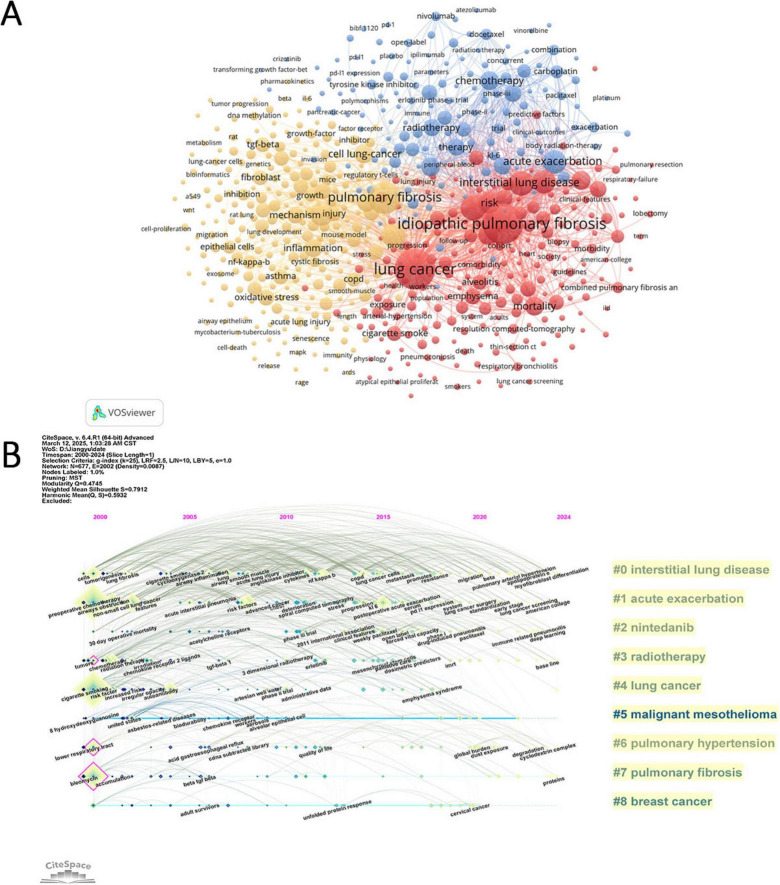
Keyword analysis in pulmonary fibrosis and lung cancer research. **(A)** Visualization of the keyword co-occurrence network in VOSviewer, displaying keywords that appeared more than five times. Node colors represent different clusters, and node size indicates frequency. **(B)** Timeline view of keyword clusters, where each horizontal line represents a cluster. Smaller numbers indicate larger clusters, with #0 being the largest. Node size reflects co-citation frequency, links represent co-citation relationships, and node appearance year indicates the first co-citation occurrence.

The co-occurrence network of keywords, visualized in Figure 7A using VOSviewer, reveals three distinct thematic clusters that reflect key research directions. The yellow cluster, enriched with terms like TGF-beta, NF-kappa-B, oxidative stress, and cigarette smoking, represents an emphasis on the molecular pathogenesis and environmental risk factors driving disease progression. This suggests ongoing efforts to elucidate inflammation pathways, epithelial-mesenchymal transition, and the impact of environmental exposures, which may contribute to both fibrosis development and carcinogenesis. The red cluster groups clinical features and diagnostic concepts, including keywords such as CT, classification, survival, risk, and hypertension. This highlights research focused on disease phenotyping, risk stratification, and understanding comorbidities that influence patient prognosis, reflecting a clinical shift toward personalized medicine approaches. The blue cluster centers on therapeutic strategies, featuring keywords like chemotherapy, immunotherapy, nivolumab, docetaxel, and pneumonitis. This cluster underscores the growing interest in immunotherapy and targeted treatments, as well as management of treatment-related adverse effects, signaling a paradigm shift in the management of PF and LC. Within the entire network, lung cancer and idiopathic pulmonary fibrosis emerge as core keywords, while highly connected nodes such as risk and expression represent key research areas. The interconnectivity among the yellow, red, and blue clusters reflects the strong interplay between disease mechanisms, clinical manifestations, and treatment approaches in the context of PF and LC. Notably, the link between lung cancer and inflammation highlights the pivotal role of inflammatory processes in tumor development, whereas the connection between lung cancer and chemotherapy underscores the ongoing emphasis on optimizing therapeutic strategies.

**FIGURE 8:**
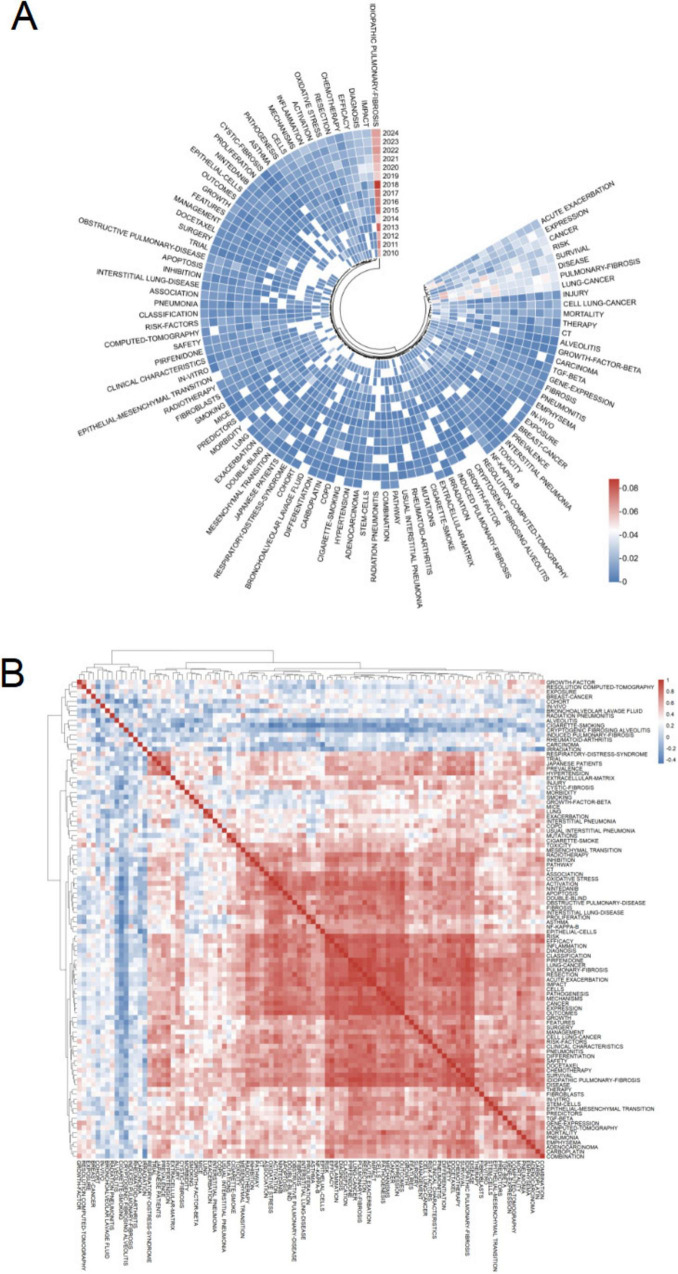
Heatmap analysis of keywords in pulmonary fibrosis and lung cancer. **(A)** Annual heatmap of keywords from 2010 to 2024. The annual heat value of each keyword is calculated by dividing the number of citations of the keyword in a given year by the total number of citations for all keywords in that year. Colors range from blue (low) to red (high), indicating variations in heat value. **(B)** Keyword correlation heatmap, illustrating the co-occurrence relationships among keywords, with color intensity representing the strength of correlation.

In CiteSpace, the timeline view captures the temporal evolution of research themes based on keyword clustering (Figure 7B). Cluster #0 (interstitial lung disease) is the earliest and most extensive, initially dominated by keywords such as “cells” and “tumorigenesis,” reflecting foundational studies on cellular behavior and cancer initiation. Recent trends in this cluster have shifted toward “pulmonary arterial hypertension,” “apolipoprotein E,” and “myofibroblast differentiation,” indicating a mechanistic focus on vascular remodeling and fibrogenesis. Cluster #1 (acute exacerbation) remains a highly active domain, with emerging keywords such as “PD-L1 expression” and “lung cancer surgery,” suggesting increasing integration of immune checkpoint research and perioperative strategies in clinical management. In contrast, clusters #5 (malignant mesothelioma) and #8 (breast cancer) have shown minimal recent activity, implying a declining relevance of these topics within the evolving landscape of PF and LC research. Figure 8A is a heatmap generated using ChiPlot^1^ (accessed on March 10, 2025). It illustrates the annual popularity of keywords from 2010 to 2024 (number of citations in the year/total citations in the year). Apart from idiopathic PF and LC, which have consistently remained research hotspots, keywords such as microenvironment, apoptosis, gene expression, radiation pneumonitis, immune checkpoint inhibitors, TGF-beta, extracellular matrix, and single-cell RNA sequencing have shown significant growth in research interest. These emerging themes underscore current research priorities focused on elucidating pathophysiological mechanisms, identifying novel therapeutic targets, and advancing precision medicine in the context of PF and LC. Furthermore, Figure 8B displays the correlations of keyword popularity. The co-occurrence of immune checkpoint inhibitors and pulmonary fibrosis suggests that the application of immunotherapy in pulmonary diseases is gaining increasing attention. Similarly, the association between radiation pneumonitis and extracellular matrix remodeling implies a growing research interest in the tissue changes induced by radiation damage. This integrated perspective helps explain the observed publication trends and points to important directions for future investigation.

### 3.7 Analysis of reference

Table 6 presents the top ten most frequently cited papers. The most frequently cited article is “Lung Cancer 2020: Epidemiology, Etiology, and Prevention” (29) (1,207 citations), which focuses on the strong relationship between the increasing incidence of lung cancer and tobacco use (29). Following this, “Acute Exacerbation of Idiopathic Pulmonary Fibrosis: An International Working Group Report” (30) (973 citations) describes acute exacerbation of idiopathic pulmonary fibrosis (AE-IPF) (30).

**TABLE 6:** Top 10 highly cited references.

Rank	Article title	Source title	Authors	Year	Cited	DOI
1	Lung cancer 2020 epidemiology, etiology, and prevention	Clinics in chest medicine	Bade BC; Dela Cruz CS	2020	1207	10.1016/j.ccm.2019.10.00
2	Acute exacerbation of idiopathic pulmonary fibrosis an international working group report	American journal of respiratory and critical care medicine	Collard HR; Ryerson C, et al.	2016	973	10.1164/rccm.201604-0801C
3	CXC chemokines in angiogenesis	Journal of leukocyte biology	Belperio JA, Keane MP, et al.	2000	648	
4	American society of Clinical Oncology clinical evidence review on the ongoing care of adult cancer survivors: Cardiac and pulmonary late effects	Journal of clinical oncology	Carver, JR; Shapiro, CL;	2007	582	10.1200/JCO.2007.10.977
5	Lung cancer in never smokers: A review	Journal of clinical oncology	Subramanian, J; Govindan, R	2007	512	10.1200/JCO.2006.06.801
6	Lung cancer and cryptogenic fibrosing alveolitis—A population-based cohort study	American journal of respiratory and critical care medicine	Hubbard, R; Venn, A;	2000	496	10.1164/ajrccm.161.1.990606
7	Properties of FDA-approved small molecule protein kinase inhibitors: A 2020 update	Pharmacological research	Roskoski, R	2020	419	10.1016/j.phrs.2019.10460
8	Lung cancer in never smokers: clinical epidemiology and environmental risk factors	Clinical cancer research	Samet, JM; Avila-Tang, E;	2009	405	10.1158/1078-0432.CCR-09-037
9	Lung cancer screening, Version 3.2018	Journal of the national comprehensive cancer network	Wood, DE; Kazerooni, EA;	2018	403	10.6004/jnccn.2018.002
10	Properties of FDA-approved small molecule protein kinase inhibitors	Pharmacological research	Roskoski, R	2019	375	10.1016/j.phrs.2019.03.00

**FIGURE 9:**
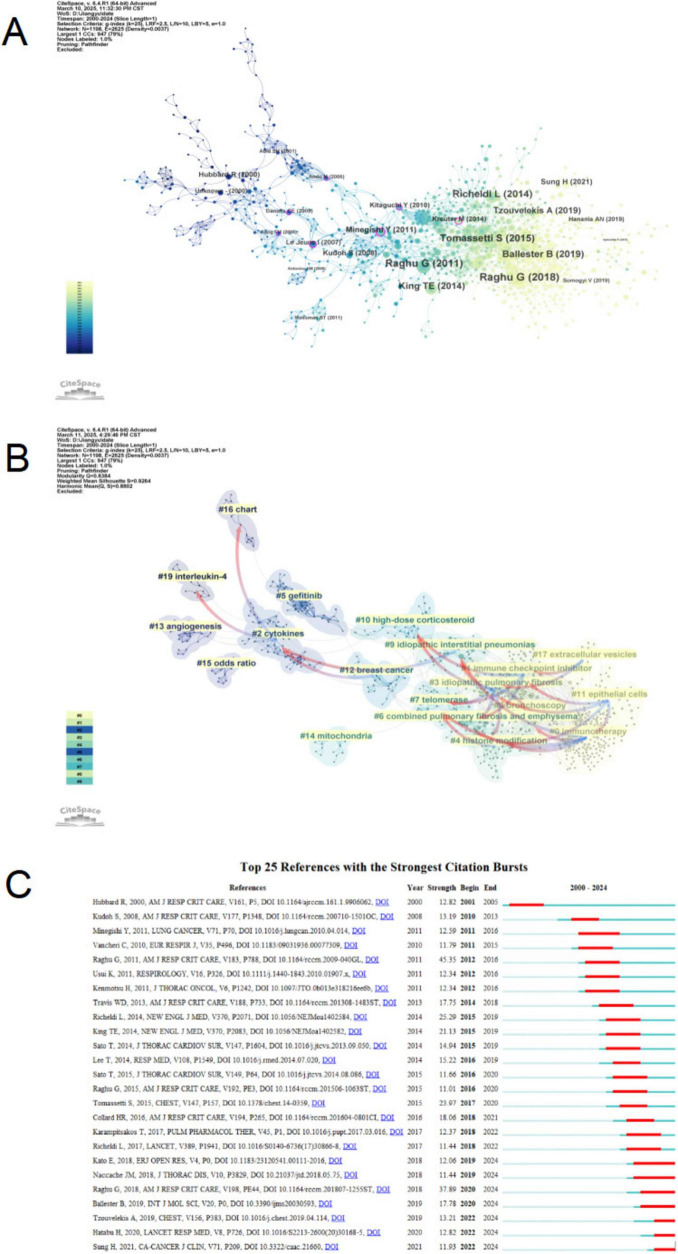
Citation analysis of pulmonary fibrosis and lung cancer. **(A)** Citation network analysis in CiteSpace. The node size is proportional to the number of times an article is co-cited. **(B)** Clustering of citations based on their similarity, each cluster represents a distinct research topic. **(C)** Top 25 references with the strongest citation bursts. Red bars represent the burst duration, indicating the time periods during which the references received a sharp increase in citations. Blue lines denote the full timeline from 2010 to 2024.

Co-citation analysis was conducted to examine relationships between articles by analyzing their co-citation frequency (31). These relationships are visualized in CiteSpace, where Figures 9A,B illustrate the authors and publication years of burst references—articles that have experienced a surge in citation frequency. Based on the degree of association among the references, clustering was performed, resulting in 19 clusters, each represented by a distinct color. The category with the highest number of publications is cluster #0 (immunotherapy). From a chronological perspective, the earliest research areas in the field of PF and LC comprised several independent research clusters, such as #13 (Angiogenesis) and #19 (Interleukin-4). These subsequently evolved into #2 (Cytokines), which later expanded into #12 (Breast cancer) and #9 (Idiopathic interstitial pneumonias). Additionally, #10 (High-dose corticosteroid), #6 (Combined pulmonary fibrosis and emphysema), and #7 (Telomerase) emerged as independent research clusters. Cluster #10 primarily developed into clusters #4 and #3, while cluster #7 also evolved into clusters #4 and #3. Cluster #6 further progressed into clusters #4 and #1. After 2016, research has increasingly focused on immunotherapy, the mechanisms underlying lung cancer coexisting with pulmonary fibrosis, and potential biomarkers. This shift has resulted in the emergence of clusters such as #0 (Immunotherapy), #1 (Immune checkpoint inhibitor), #6 (Combined pulmonary fibrosis and emphysema), and #9 (Idiopathic interstitial pneumonias).

Figure 9A also calculates the betweenness centrality of nodes, revealing that Kong (32), Daniels (33), Ando (34), Le Jeune (35), Minegishi (36), Kitaguchi (37), and Kreuter (38) possess high betweenness centrality, indicating that these articles serve as crucial links between different research phases. Figure 9C displays the top 25 most frequently cited references. Two citation bursts occurred before 2010, corresponding to the articles “Lung Cancer and Cryptogenic Fibrosing Alveolitis—A Population-Based Cohort Study” and “Interstitial Lung Disease in Japanese Patients with Lung Cancer—A Cohort and Nested Case-Control Study.” Additionally, “An Official ATS/ERS/ARS/ALAT Statement: Idiopathic Pulmonary Fibrosis: Evidence-Based Guidelines for Diagnosis and Management” experienced a significant citation burst in 2012 (burst strength = 45.35). Similarly, “Diagnosis of Idiopathic Pulmonary Fibrosis: An Official ATS/ERS/JRS/ALAT Clinical Practice Guideline” showed a notable citation burst in 2020 (burst strength = 37.89). Apart from these two official guidelines, “Efficacy and Safety of Nintedanib in Idiopathic Pulmonary Fibrosis” exhibited a high citation burst from 2015 to 2019 (burst strength = 25.29). The results indicate that 2016, 2018, and 2019 each witnessed four citation bursts, suggesting that the period from 2016 to 2019 was a crucial phase of heightened research interest and rapid growth in this field. Moreover, the citation bursts of “Global Cancer Statistics 2020: GLOBOCAN Estimates of Incidence and Mortality Worldwide for 36 Cancers in 185 Countries” (39) and “Interstitial Lung Abnormalities Detected Incidentally on CT: A Position Paper from the Fleischner Society” (40) have persisted until 2024, indicating that research in this domain remains active and ongoing.

### 3.8 Subject area analysis

The CiteSpace analysis reveals the citation relationships of PF and LC-related literature categorized by disciplinary fields (Supplementary Figure 3). Publications associated with the “RESPIRATORY SYSTEM” discipline received the highest number of citations, followed by “ONCOLOGY,” “PHARMACOLOGY & PHARMACY,” “BIOCHEMISTRY & MOLECULAR BIOLOGY,” “RADIOLOGY, NUCLEAR MEDICINE & MEDICAL IMAGING,” “CELL BIOLOGY,” “MEDICINE, RESEARCH & EXPERIMENTAL,” “MEDICINE, GENERAL & INTERNAL,” “SURGERY,” and “CARDIAC & CARDIOVASCULAR SYSTEMS.” Disciplines including “BIOCHEMISTRY & MOLECULAR BIOLOGY,” “ENGINEERING, ELECTRICAL & ELECTRONIC,” “RESPIRATORY SYSTEM,” “PHARMACOLOGY & PHARMACY”, “PUBLIC, ENVIRONMENTAL & OCCUPATIONAL HEALTH”, “CELL BIOLOGY,” “RADIOLOGY, NUCLEAR MEDICINE & MEDICAL IMAGING,” “ONCOLOGY,” “MEDICINE, RESEARCH & EXPERIMENTAL,” “CHEMISTRY, MULTIDISCIPLINARY”, “NANOSCIENCE & NANOTECHNOLOGY,” “BIOCHEMICAL RESEARCH METHODS,” “ENGINEERING, BIOMEDICAL,” and “TOXICOLOGY” are highlighted with purple rings, indicating their greater influence in this research field. The impact of these disciplines is represented by the presence of purple rings surrounding them.

## 4 Discussion

CiteSpace 6.4.R1 Advanced, VOSviewer 1.6.20, and R-bibliometrics were utilized to analyze data from 1,830 articles on PF and LC retrieved from the Web of Science between 2000 and 2024. Based on these data, the study evaluates the spatiotemporal distribution, author contributions, core articles, research hotspots, and emerging trends in this field.

### 4.1 General distribution

The analysis in this study is based on 1,830 articles related to PF and LC retrieved from the Web of Science Core Collection (WOSCC) database between January 1, 2000, and December 31, 2024. The rapid growth in the number of publications indicates increasing attention to PF and LC. Over the past decade, research on these topics has shown an upward trend, with the number of publications in 2024 being more than three times that of 2013.

In the country/region analysis, the two most critical indicators are the number of publications and betweenness centrality. High betweenness centrality (≥ 0.10) signifies that these countries/regions act as “bridges” in the global collaboration network. As shown in Tables 1, 2, and Figures 3, 4, the United Kingdom, Canada, the United States, Australia, Italy, and Germany are the central countries in PF and LC research. The United States has the highest citation frequency and ranks second in publication count. The United Kingdom ranks seventh in publication count and fourth in citation frequency. Canada ranks eighth in publication count and tenth in citation frequency. Australia ranks tenth in publication count and ninth in citation frequency. Italy ranks fifth in publication count and sixth in citation frequency. Germany ranks fourth in publication count and fifth in citation frequency.

Among the top 10 institutions by publication count, four are from Japan, followed by two from the United States and two from China. Among the top ten institutions by citation frequency, six are from the United States. Ulsan University in South Korea ranks among the top ten in both publication count and citation frequency. The United Kingdom has the highest centrality (0.33) among all countries, followed by Canada (0.21), indicating its dominant role in global research collaboration on PF and LC. Additionally, China, Japan, South Korea, and France have actively participated in research and collaboration on this topic.

As shown in Tables 3, 6, and Supplementary Table 4, Suzuki Kenji is the most prolific author. Notably, Bade BC, who ranks sixth in citation frequency, authored the highly cited article “Lung Cancer 2020: Epidemiology, Etiology, and Prevention,” which ranks first in citation frequency and has made a significant impact in the field.

As presented in Table 4 and Figure 5, the International Journal of Radiation Oncology Biology Physics and PLOS ONE are not only among the top 10 journals by publication count but also among the top 10 co-cited journals, primarily due to their publication of numerous highly cited articles. Notably, among the top 10 co-cited journals, five are medical journals (N Engl J Med, Chest, Thorax, Eur Respir J, Am J Respir Crit Care Med), excluding multidisciplinary journals. Two are molecular biology-related journals (J Biol Chem, Am J Respir Cell Mol Biol), and Int J Radiat Oncol Biol Phys and N Engl J Med are associated with clinical research, which aligns with the dual-map overlay analysis in Figure 6C.

### 4.2 Hotspots and frontiers

Keyword analysis helps in understanding the research frontiers and hot topics in the field of PF and LC. In existing studies, the primary keywords include “lung cancer,” “idiopathic pulmonary fibrosis,” “survival,” “acute exacerbation,” “risk,” “diagnosis,” “chemotherapy,” “mortality,” and “inflammation” (Table 5). These keywords are mainly related to the diagnosis, mechanisms, and prognosis of PF and LC, indicating that they represent hot topics in this research area.

The co-occurrence network analysis reveals several major research directions based on high-frequency keywords from previous studies, including etiology (e.g., NF-kappa-B, asbestos, cigarette smoking, air pollution), diagnosis and prognosis (e.g., mortality, hypertension, prevalence), and therapeutic approaches (e.g., therapy, chemotherapy, risk factors, pneumonitis, exacerbation).

Correspondingly, the timeline analysis indicates that clusters #0 (interstitial lung disease), #1 (acute exacerbation), #2 (nintedanib), and #3 (radiotherapy) are relatively large, suggesting their high research significance. As shown in Figure 7, in recent years, idiopathic pulmonary fibrosis, lung cancer, epithelial-mesenchymal transition, fibroblasts, transforming growth factor-β (TGF-β), radiation pneumonitis, immune checkpoint inhibitors, and TGF-β have remained active research areas, each representing an emerging research direction.

The rising research trend in novel therapeutic approaches is particularly notable, with increasing focus on immunotherapy, radiation-induced lung injury, and prognosis-related studies, highlighting the ongoing evolution of this field.

### 4.3 Pulmonary fibrosis and lung cancer

This study provides a comprehensive bibliometric analysis that systematically maps the research hotspots and developmental trends in PF and LC. By integrating keyword and cluster analyses with current molecular and clinical insights, we further explore the underlying pathophysiological mechanisms driving this comorbidity. Key signaling pathways such as Wnt/β-catenin, TGF-β, and PI3K/Akt, along with genetic and epigenetic alterations, not only contribute to the progression of fibrosis and tumorigenesis but also play crucial roles in shaping the immune microenvironment and therapeutic responses. The frequent emergence of terms including “immunotherapy,” “chemotherapy,” and “acute exacerbation” in the bibliometric data reflects the increasing research focus on precision diagnosis and treatment strategies for this complex disease entity.

Pulmonary fibrosis (PF) and lung cancer (LC) are both severe respiratory diseases with poor prognoses. Lung cancer associated with pulmonary fibrosis has been classified into several subtypes in the literature, mainly including idiopathic pulmonary fibrosis (UIP/IPF)-associated lung cancer, smoking-related interstitial lung disease (SR-ILD)-associated lung cancer, and other interstitial lung disease (O-ILD)-associated lung cancer (41). UIP/IPF-associated lung cancer frequently develops in peripheral fibrotic areas of the lung, typically exhibiting advanced fibrosis features such as honeycombing, with adenocarcinoma and squamous cell carcinoma as predominant histological types. The carcinogenesis is closely related to the pro-oncogenic factors in the fibrotic microenvironment. SR-ILD-associated lung cancer more often arises in the central airways, with tumor origin linked to airway epithelial injury and chronic inflammation. The tumor histology is more diverse and may be independent of fibrosis progression. O-ILD-associated lung cancer tends to occur in fibrotic or inflammatory areas, predominantly presenting as adenocarcinoma, and patients generally have a better prognosis. Overall, lung cancer subtypes associated with different pulmonary fibrosis patterns show distinct characteristics in tumor location, pathology, and oncogenic mechanisms, highlighting the close relationship between lung cancer development and the pathological features and inflammatory microenvironment of pulmonary fibrosis. This study is the first systematic bibliometric analysis of research on PF and LC from 2000 to 2024, including both original articles and reviews. It provides a comprehensive overview of the development trajectory and research hotspots in this interdisciplinary field.

Keyword analysis identified “idiopathic pulmonary fibrosis” (711) and “lung cancer” (710) as the core terms, followed by “survival,” “acute exacerbation,” “chemotherapy,” “risk,” and “inflammation.” These keywords indicate a primary focus on diagnosis, prognosis assessment, and therapeutic interventions. Consistent with these findings, a recent multicenter phase III clinical trial demonstrated that nintedanib combined with carboplatin plus albumin-bound paclitaxel effectively preserves lung function and may improve survival outcomes in NSCLC patients with IPF, particularly those with lower GAP scores (42). Although the incidence of moderate-to-severe neutropenia was slightly higher than in non-IPF patients, overall tolerability was favorable without new safety signals. This clinical evidence supports the feasibility of antifibrotic therapy combined with chemotherapy and validates high-frequency keywords such as “chemotherapy,” “survival,” and “inflammation.”

The CiteSpace timeline view (Figure 7B) reveals the evolution and shifting hotspots of research themes in PF and LC. Cluster #0 (interstitial lung diseases) represents the earliest and largest research focus, initially emphasizing basic mechanisms like “cells” and “tumorigenesis.” Recent keywords have shifted toward micro-mechanisms and potential targets such as “pulmonary arterial hypertension,” “myofibroblast differentiation,” and “apolipoprotein E.” Cluster #1 (acute exacerbation) primarily addresses clinical management strategies. The emergence of terms like “PD-L1 expression” and “lung cancer surgery” indicates growing interest in the efficacy and safety of immune checkpoint inhibitors (ICIs) in surgical patients. Overall, a positive interaction between basic and clinical research is evident, with the potential role of immunotherapy in acute exacerbations increasingly recognized.

According to the clustering analysis shown in Figure 9B, growing research interest has centered on pathogenic mechanisms and treatment approaches. Key clusters include #0 (immunotherapy), #1 (immune checkpoint inhibitors), #11 (epithelial cells), and #17 (extracellular vesicles). The pathogenesis of the association between PF and LC is not well understood. Potential mechanisms include epigenetic and genetic alterations in the lung as a result of chronic inflammation induced by smoking and the underlying dysregulated fibrotic process (14, 43). Environmental risk factors such as smoking exposure and aging, which are commonly observed in both PF and LC patients, have been associated with epigenetic alterations, including changes in DNA methylation patterns, as revealed by genome-wide methylation analysis (44–46). Notably, hypermethylation of the CD90/Thy-1 promoter suppresses its expression, enhancing cancer cell invasiveness and fibrosis progression (47, 48). In addition to epigenetic modifications, specific gene mutations play a crucial role in the initiation and progression of both PF and LC (49). Mutations in surfactant protein genes SFTPA1 and SFTPA2 in lung adenocarcinoma impair protein secretion, induce endoplasmic reticulum stress and apoptosis, fostering co-occurrence of PF and LC (50, 51).

Another potential mechanism is the dysregulation of multiple interrelated signaling pathways (14, 52). Recent single-cell RNA sequencing (scRNA-seq) studies have revealed the critical role of aberrant cell–cell communication and senescence-associated pathways in therapy-induced lung injury, a condition frequently associated with both fibrosis and cancer. In a murine model combining lung irradiation and anti-PD-1 immunotherapy, accumulation of senescence-like fibroblasts, macrophages, and alveolar epithelial cells was observed, accompanied by disrupted intercellular signaling and extracellular matrix remodeling (53). These findings underscore the emerging importance of cellular senescence and microenvironmental dysregulation in the pathogenesis of fibrotic and tumorigenic pulmonary diseases. The Wnt/β-catenin pathway, which regulates cell proliferation, differentiation, and extracellular matrix (ECM) remodeling, is aberrantly activated in both diseases, contributing to fibrotic progression and tumorigenesis (54). Notably, this pathway extensively crosstalks with transforming growth factor-beta (TGF-β), a central mediator in fibrosis and malignancy, promoting fibroblast-to-myofibroblast differentiation, ECM deposition, and epithelial-mesenchymal transition (EMT), thereby facilitating tumor invasion and metastasis (54, 55). Furthermore, scRNA-seq analyses of EGFR-mutant non-small cell lung cancer (NSCLC) during targeted therapy reveal dynamic alterations in key signaling pathways and the immune microenvironment (56). Cancer cells at the residual disease (RD) stage show pronounced activation of the Wnt/β-catenin pathway, linked to cellular regeneration and phenotypic plasticity. This stage also features increased infiltration of T lymphocytes and an immunologically active tumor microenvironment, suggesting a transient window of immune responsiveness. The PI3K/Akt pathway sustains fibroblast activity and resists apoptosis in both PF and LC (54, 57). Key fibrogenic and oncogenic factors include TGF-β, PDGF, VEGF, and FGF; among these, VEGF activates ERK1/2 and PI3K pathways to promote proliferation (54, 58–60). Fibrosis exacerbation associates with immune and structural microenvironment alterations, fostering an immunosuppressive tumor microenvironment (TME). Sustained activation of TGF-β, PI3K/Akt, and Wnt pathways modulates regulatory T cells, induces M2 macrophages, and suppresses cytotoxic T lymphocytes, facilitating immune evasion. Targeting these pathways and modulating the TME may offer novel therapeutic strategies for PF and LC, potentially reversing immune suppression and improving outcomes (61–63).

Preexisting interstitial lung disease (ILD), particularly idiopathic pulmonary fibrosis (IPF), is a well-established risk factor for checkpoint inhibitor-related pneumonitis (CIP). In lung cancer patients receiving immune checkpoint inhibitor (ICI) therapy, the presence of ILD significantly increases the likelihood of developing CIP (64–66). However, recent research has developed a biomimetic ICI protein (EMS-PDBP) that specifically targets intracellular PD-L1 and leverages erythrocyte membrane encapsulation to enhance selective uptake in cancer cells, thereby minimizing its effects on lung fibroblasts. This strategy successfully restored antitumor immunity in a LUAD-IPF mouse model while significantly reducing irAEs, offering a novel approach for the immunotherapy of lung cancer with coexisting pulmonary fibrosis (67).

Based on a systematic bibliometric analysis, this study maps the current research hotspots and developmental trends in PF and LC. It further proposes potential future directions, such as elucidating the inflammation–fibrosis–carcinogenesis continuum and identifying key molecular targets, as well as optimizing combined immunotherapy and antifibrotic treatment strategies. These perspectives may help guide future basic and clinical investigations in this field.

## 5 Conclusion

This bibliometric study provides a comprehensive overview of the research landscape and collaborative networks in PF and LC over the past 25 years. The field has evolved from focusing mainly on diagnosis and prognosis to emphasizing molecular mechanisms and immunotherapy, reflecting growing insights into disease interactions. Key research areas now include epithelial-mesenchymal transition, chronic inflammation, and immune checkpoint inhibitors. Our findings highlight these topics as current priorities and reveal gaps that require further exploration, particularly regarding immune-related pathways and novel biomarkers. Future research should aim to validate molecular targets and evaluate the clinical efficacy of immunotherapy in patients with both PF and LC. Enhancing international collaboration will be crucial to accelerate advancements in this complex area. Overall, this study offers valuable guidance for researchers to better understand ongoing trends and identify promising directions for future work.

## 6 Limitations

This study is the first to apply bibliometric visualization techniques to analyze the research landscape of pulmonary fibrosis and lung cancer over the past 25 years. However, several limitations should be acknowledged. First, the analysis was exclusively based on data from the Web of Science Core Collection (WOSCC), excluding other databases such as PubMed, Cochrane Library, and Google Scholar. Although WOSCC is a widely used and comprehensive database, its coverage is not exhaustive, which may lead to potential selection bias. Second, only English-language publications were included, which could further limit the comprehensiveness of the retrieved literature and exclude relevant studies published in other languages. Third, bibliometric tools such as CiteSpace and VOSviewer rely on citation counts and keyword co-occurrence, which are subject to inherent biases. Highly cited papers may disproportionately influence clustering outcomes, while keyword-based analyses can be affected by inconsistencies in terminology, such as abbreviations, synonyms, and spelling variations. Despite efforts to clean and standardize the data, such inconsistencies may lead to fragmented clusters or distort thematic interpretation. Furthermore, the process of cluster labeling is partly subjective and may not fully capture the nuances of research content. Lastly, the data may contain discrepancies due to institutional name changes or inconsistencies in author affiliations over time, which can affect the accuracy of collaboration network analyses. Given these limitations, the findings should be interpreted with caution and considered as reflective of research trends rather than conclusive evidence of scientific impact or clinical effectiveness.

## Data Availability

The raw data supporting the conclusions of this article will be made available by the authors, without undue reservation.

## Footnotes

1
https://www.chiplot.online/
