# Supplementary material for: Advances and frontiers in pulmonary fibrosis and lung cancer research (2000–2024): a bibliometric analysis
Source: Front Med (Lausanne). 2025 Jun 23;12:1596228. doi: 10.3389/fmed.2025.1596228 (PMC12229879; doi:10.3389/fmed.2025.1596228)
Supplement: Supplementary file 1 [file Data_Sheet_1.docx]

Supplementary Material

## Supplementary **Figure**s

**Supplementary Figure 1** Analysis of the collaboration network of countries/regions in CiteSpace.


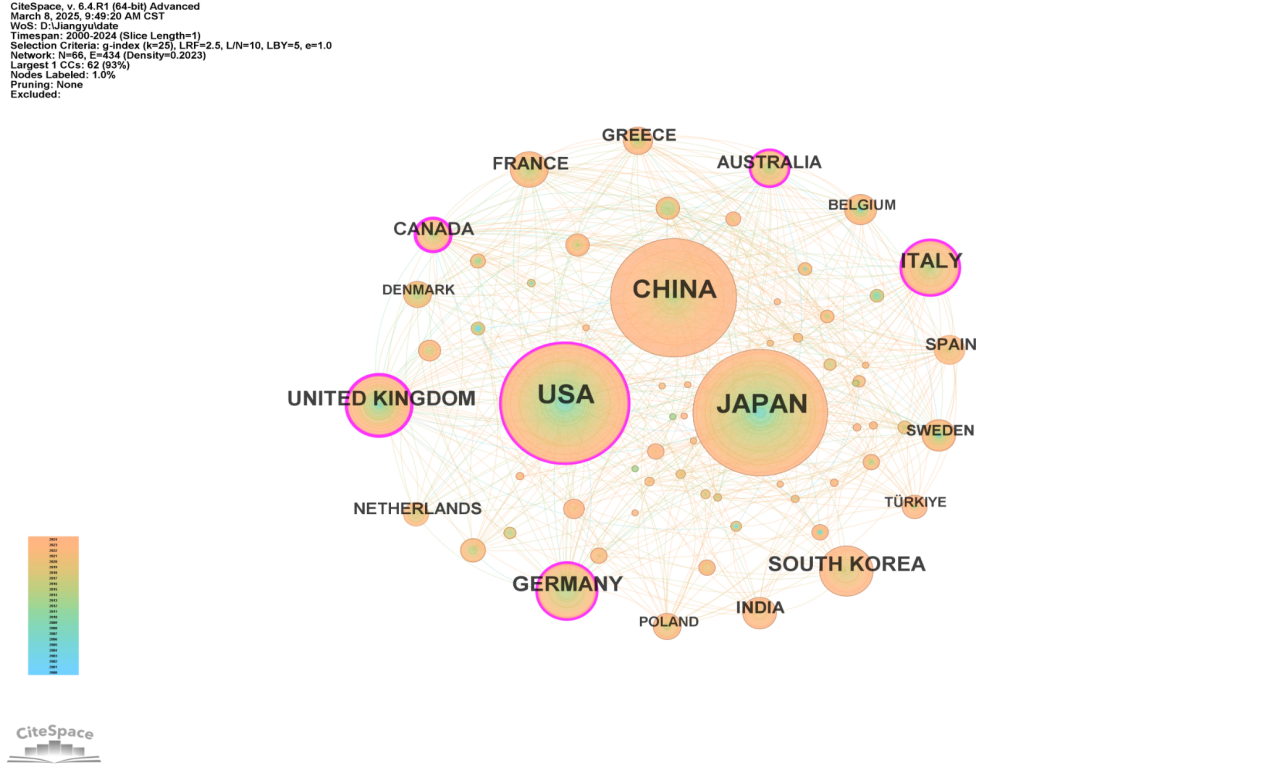


**Supplementary Figure 1** shows collaboration network of countries/regions. The size of the nodes indicates the co-occurrence frequencies, and the links indicate the co-occurrence relationship. The nodes with purple outer circles represent their higher centrality.

**Supplementary Figure 2** Analysis of collaborative network visualization of institutions in CiteSpace.


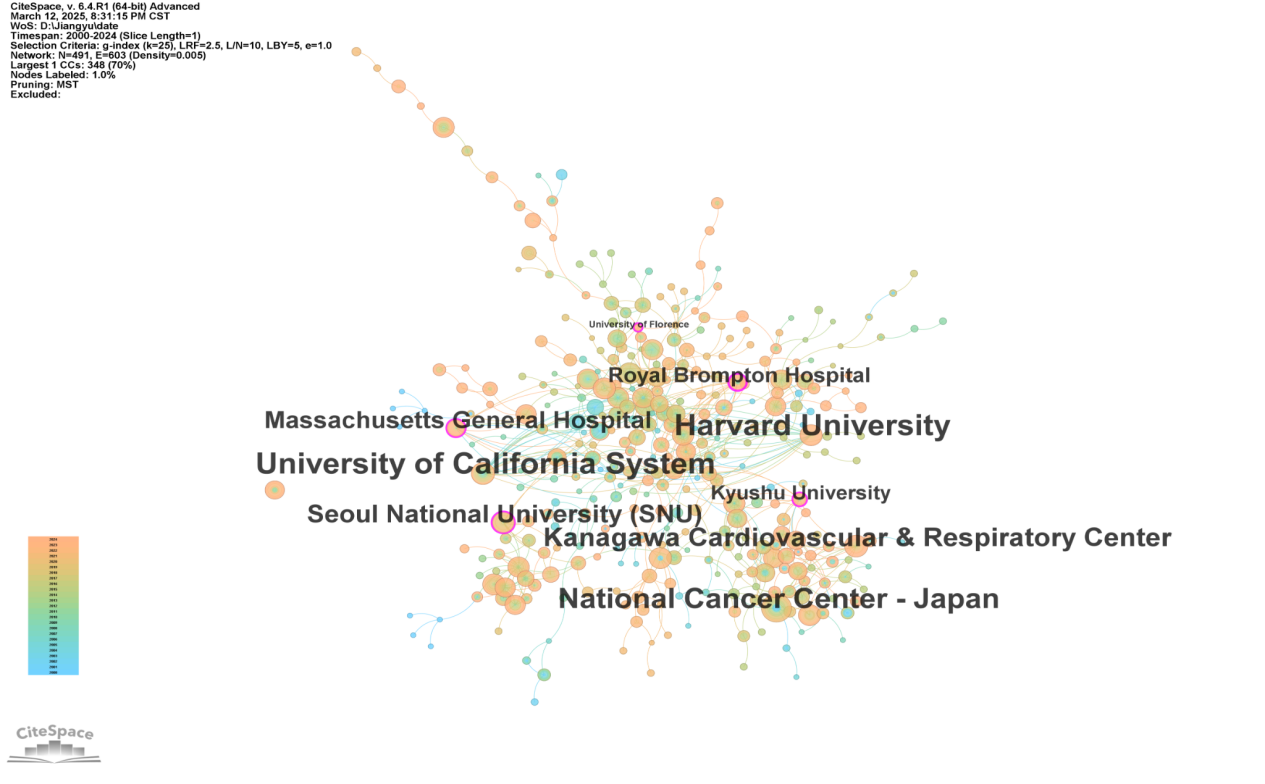
**Supplementary Figure 2** shows collaborative network visualization of institutions. The size of the nodes indicates the co-occurrence frequencies, the links indicate the co-occurrence relationship, and the nodes with purple outer circles represent their higher centrality.

**Supplementary Figure 3**Analysis of Pulmonary Fibrosis and Lung Cancer correlation subject.


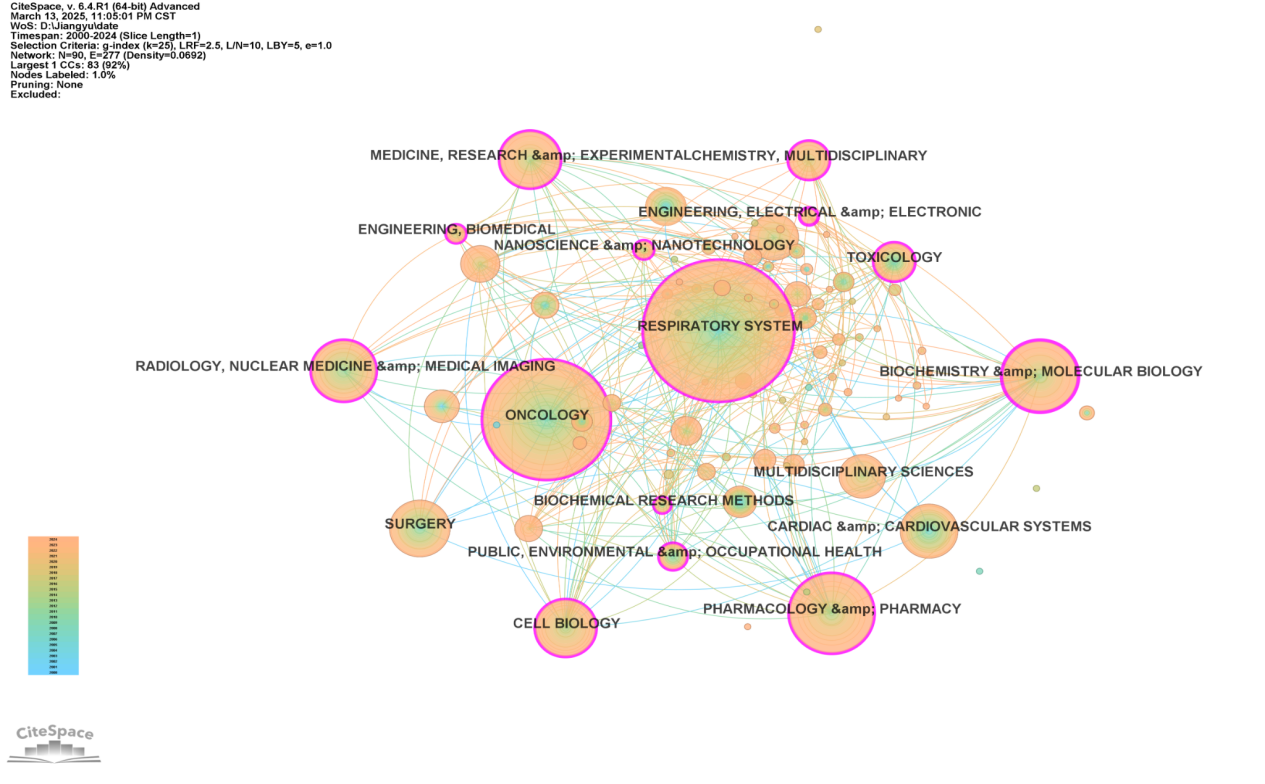


**Supplementary Figure 3** shows the analysis of Pulmonary Fibrosis and Lung Cancer correlation subject. The size of the nodes indicates the co-occurrence frequencies, the links indicate the co-occurrence relationship, and the nodes with purple outer circles represent their higher centrality.

## 2. Supplementary Tables

**Supplementary Table 1** Top 10 institutions in terms of centrality.

| Rank | Institution | Publications | Centrality |
| --- | --- | --- | --- |
| 1 | Royal Brompton Hospital | 11 | 0.14 |
| 2 | Assistance Publique Hopitaux Paris (APHP) | 22 | 0.11 |
| 3 | Seoul National University (SNU) | 20 | 0.11 |
| 4 | Harvard University | 39 | 0.09 |
| 5 | University of California System | 38 | 0.09 |
| 6 | George Papanikolaou General Hospital of Thessaloniki | 3 | 0.09 |
| 7 | Nippon Medical School | 17 | 0.08 |
| 8 | Imperial College London | 15 | 0.08 |
| 9 | Ruprecht Karls University Heidelberg | 19 | 0.08 |
| 10 | Massachusetts General Hospital | 17 | 0.07 |

**Supplementary Table 2** Top 10 authors in terms of number of frequency of co-citations

| Rank | Author | Citations | Total link strength |
| --- | --- | --- | --- |
| 1 | raghu g | 988 | 25000 |
| 2 | cottin v | 343 | 11295 |
| 3 | richeldi l | 319 | 8457 |
| 4 | king te | 298 | 8096 |
| 5 | hubbard r | 262 | 5520 |
| 6 | sato t | 248 | 5427 |
| 7 | collard hr | 234 | 6061 |
| 8 | minegishi y | 210 | 5154 |
| 9 | travis wd | 209 | 4210 |
| 10 | vancheri c | 201 | 5524 |

**Supplementary Table 3** Top 10 journals in terms of number of frequency of co-citations, corresponding IF (JCR2023) and JCR quartile.

| Rank | Journal | IF (JCR2023) | JCR quartile | Co-citations | Total link strength |
| --- | --- | --- | --- | --- | --- |
| 1 | am j resp crit care | 19.3 | Q1 | 5260 | 410550 |
| 2 | eur respir j | 17 | Q1 | 2697 | 233733 |
| 3 | chest | 9.5 | Q1 | 2628 | 212216 |
| 4 | new engl j med | 96.3 | Q1 | 1917 | 163819 |
| 5 | plos one | 2.9 | Q1 | 1615 | 172482 |
| 6 | int j radiat oncol | 6.4 | Q1 | 1511 | 68302 |
| 7 | am j resp cell mol | 5.9 | Q1 | 1392 | 155949 |
| 8 | thorax | 10.8 | Q1 | 1371 | 121700 |
| 9 | p natl acad sci usa | 9.4 | Q1 | 1253 | 135159 |
| 10 | j biol chem | 4.0 | Q2 | 1222 | 126806 |

**Supplementary Table 4** Top 10 authors in terms of frequency of citations.

| Rank | Author | Documents | Citations | Total link strength |
| --- | --- | --- | --- | --- |
| 1 | burdick, md | 9 | 1985 | 46 |
| 2 | belperio, ja | 8 | 1863 | 41 |
| 3 | strieter, rm | 8 | 1807 | 40 |
| 4 | keane, mp | 7 | 1760 | 34 |
| 5 | eickelberg, oliver | 10 | 1235 | 58 |
| 6 | bade, brett c. | 1 | 1207 | 1 |
| 7 | dela cruz, charles s. | 1 | 1207 | 1 |
| 8 | kreuter, michael | 15 | 850 | 128 |
| 9 | roskoski, robert, jr. | 2 | 794 | 0 |
| 10 | lee, kyung soo | 7 | 765 | 62 |
